# Multisensory integration in action control

**DOI:** 10.3389/fpsyg.2014.00544

**Published:** 2014-06-10

**Authors:** Christine Sutter, Knut Drewing, Jochen Müsseler

**Affiliations:** ^1^Department of Work and Cognitive Psychology, RWTH Aachen UniversityAachen, Germany; ^2^Department for Experimental Psychology, Institute for Psychology, Justus-Liebig UniversityGiessen, Germany

**Keywords:** human information processing, perception, tool use, recalibration, reference frame, vision, haptic, acoustics

The integration of multisensory information is an essential mechanism in perception and action control. Research in multisensory integration is concerned with how the information from the different sensory modalities, such as the senses of vision, hearing, smell, taste, touch, and proprioception, are integrated to a coherent representation of objects (for an overview, see e.g., Calvert et al., 2004). The combination of information from the different senses is central for action control. For instance, when you grasp for a rubber duck, you can see its size, feel its compliance and hear the sound it produces. Moreover, identical physical properties of an object can be provided by different senses. You can both see and feel the size of the rubber duck. Even when you grasp for the rubber duck with a tool (e.g., with tongs), the information from the proximal hand, from the effective part of the distal tool and from the eyes are integrated in a manner to act successfully (for limitations of this integration see Sutter et al., 2013).

Over the recent decade a surge of interest in multisensory integration and action control has been witnessed, especially in connection with the idea of a statistically optimized integration of multiple sensory sources. The human information processing system is assumed to adjust moment-by-moment the relative contribution of each sense's estimate to a multisensory task. The sense's contribution depends on its variance, so that the total variance of the multisensory estimate is lower than that for each sense alone. Accordingly, the validity of a statistically optimized multisensory integration has been demonstrated by extensive empirical research (e.g., Ernst and Banks, 2002; Alais and Burr, 2004; Reuschel et al., 2010), also in applied setting such as tool-use (e.g., Takahashi et al., 2009; in the present research topic: Takahashi and Watt, 2014).

For this perspective to mature it will be helpful to delve deeper into the multisensory information processing mechanisms and their neural correlates, asking about the range and constraints of these mechanisms, about its localization and involved networks. The contributions to the present research topic range from how information from different senses and action control are linked and modulated by object affordances (Garrido-Vásquez and Schubö, 2014), by task-irrelevant information (Juravle et al., 2013; Wendker et al., 2014; for a review see Wesslein et al., 2014), by temporal and spatial coupling within and between senses (Cameron et al., 2014; Mueller and Fiehler, 2014; Rieger et al., 2014; Sugano et al., 2014) to childhood development of multisensory mechanisms (Jovanovic and Drewing, 2014).

Correspondences between the information from different senses play an important role for multisensory integration. Integration does, for instance, not take place when vision and touch are spatially separated (e.g., Gepshtein et al., 2005). However, cognitive approaches on action effect control assume that information from different senses is still coded and represented within the same cognitive domain, when the information concerns the same action (e.g., Müsseler, 1999; Hommel et al., 2001). The present research topic also addresses the corresponding issue of modality-specific action control (Boutin et al., 2013; Grunwald et al., 2014).

Overall, the present research topic broadens our view on how multisensory mechanisms add to action control. We thank all authors and all reviewers for their valuable contributions.

## Conflict of interest statement

The authors declare that the research was conducted in the absence of any commercial or financial relationships that could be construed as a potential conflict of interest.

## References

[B1] Alais D. Burr D. (2004). The ventriloquist effect results from near-optimal bimodal integration. Curr. Biol. 14, 257–262 10.1016/j.cub.2004.01.029 14761661

[B2] Boutin A. Massen C. Heuer H. (2013). Modality-specific organization in the representation of sensorimotor sequences. Front. Psychol. 4:937 10.3389/fpsyg.2013.00937 24376432 PMC3858678

[B3] Calvert G. A. Spence C. Stein B. E. (2004). The Handbook of Multisensory Processes. Cambridge, MA: The MIT Press

[B4] Cameron B. D. de la Malla C. López-Moliner J. (2014). The role of differential delays in integrating transient visual and proprioceptive information. Front. Psychol. 5:50 10.3389/fpsyg.2014.00050 24550870 PMC3910305

[B5] Ernst M. O. Banks M. S. (2002). Humans integrate visual and haptic information in a statistically optimal fashion. Nature 415, 429–433 10.1038/415429a 11807554

[B6] Garrido-Vásquez P. Schubö A. (2014). Modulation of visual attention by object affordance. Front. Psychol. 5:59 10.3389/fpsyg.2014.00059 24567725 PMC3915415

[B7] Gepshtein S. Burge J. Ernst M. O. Banks M. S. (2005). The combination of vision and touch depends on spatial proximity. J. Vis. 5, 1013–1023 10.1167/5.11.7 16441199 PMC2632311

[B8] Grunwald M. Muniyandi M. Kim H. Kim J. Krause F. Mueller S. (2014). Human haptic perception is interrupted by explorative stops of milliseconds. Front. Psychol. 5:292 10.3389/fpsyg.2014.00292 24782797 PMC3988394

[B9] Hommel B. Müsseler J. Aschersleben G. Prinz W. (2001). The theory of event coding (TEC): a framework for perception and action. Behav. Brain Sci. 24, 869–937 10.1017/S0140525X01000103 12239891

[B10] Jovanovic B. Drewing K. (2014). The influence of intersensory discrepancy on visuo-haptic integration is similar in 6-year-old children and adults. Front. Psychol. 5:57 10.3389/fpsyg.2014.00057 24523712 PMC3906500

[B11] Juravle G. McGlone F. Spence C. (2013). Context-dependent changes in tactile perception during movement execution. Front. Psychol. 4:913 10.3389/fpsyg.2013.00913 24367346 PMC3853591

[B12] Mueller S. Fiehler K. (2014). Gaze-dependent spatial updating of tactile targets in a localization task. Front. Psychol. 5:66 10.3389/fpsyg.2014.00066 24575060 PMC3918658

[B13] Müsseler J. (1999). How independent from action control is perception? An event-coding account for more equally-ranked crosstalks. Adv. Psychol. 129, 121–147 10.1016/S0166-4115(99)80014-4

[B14] Reuschel J. Drewing K. Henriques D. Y. P. Rösler F. Fiehler K. (2010). Optimal integration of visual and proprioceptive movement information along angular trajectories. Exp. Brain Res. 201, 853–862 10.1007/s00221-009-2099-4 19953230

[B15] Rieger M. Dietrich S. Prinz W. (2014). Effects of angular gain transformations between movement and visual feedback on coordination performance in unimanual circling. Front. Psychol. 5:152 10.3389/fpsyg.2014.00152 24634665 PMC3942634

[B16] Sugano Y. Keetels M. Vroomen J. (2014). Concurrent sensorimotor temporal recalibration to different lags for the left and right hand. Front. Psychol. 5:140 10.3389/fpsyg.2014.00140 24624098 PMC3934310

[B17] Sutter C. Sülzenbrück S. Rieger M. Müsseler J. (2013). Limitations of distal effect anticipation when using tools. New Ideas Psychol. 31, 247–257 10.1016/j.newideapsych.2012.12.001

[B18] Takahashi C. Diedrichsen J. Watt S. J. (2009). Integration of vision and haptics during tool use. J. Vis. 9, 3.1–3.13 10.1167/9.6.3 19761294

[B19] Takahashi C. Watt S. J. (2014). Visual-haptic integration with pliers and tongs: signal weights take account of changes in haptic sensitivity caused by different tools. Front. Psychol. 5:109 10.3389/fpsyg.2014.00109 24592245 PMC3924038

[B20] Wendker N. Sack O. S. Sutter C. (2014). Visual target distance, but not visual cursor path length produces shifts in motor behavior. Front. Psychol. 5:225 10.3389/fpsyg.2014.00225 24672507 PMC3956313

[B21] Wesslein A.-K. Spence C. Frings C. (2014). Vision affects tactile target and distractor processing even when space is task-irrelevant. Front. Psychol. 5:84 10.3389/fpsyg.2014.00084 24567727 PMC3915095

